# A stereo dataset of annotated budgerigar flight trajectories for multi-agent collision avoidance studies

**DOI:** 10.1016/j.dib.2026.112956

**Published:** 2026-06-09

**Authors:** S.M. Tawhid, Abdul Kader Mohim, Sk. Shahed Ali, Kazi Tanzizul Haque Tanzil, Dip Nandi, Abhijit Bhowmik, Debajyoti Karmaker

**Affiliations:** Department of Computer Science, American International University-Bangladesh (AIUB), Kuratoli, Khilkhet, Dhaka 1229, Bangladesh

**Keywords:** 3D reconstruction, Multi-object tracking, Animal behaviour analysis, Computer vision, Pose estimation, Flight interaction analysis, Stereo triangulation

## Abstract

This article presents a collection of 360 synchronized stereo video pairs capturing the flight behaviour of Budgerigars (*Melopsittacus undulatus*) in a controlled indoor arena. The recordings were acquired at 120 frames per second using a calibrated stereo camera setup with a fixed baseline. The dataset includes both solo flight sequences and structured head-on interaction scenarios involving one-to-one, two-to-two, and three-to-three group configurations. All individuals are manually annotated in both camera views using identity-consistent bounding boxes along with four body keypoints: head, tail, left wing, and right wing. In total, the dataset contains 2760,178 labelled annotations. The synchronized two-dimensional annotations from both views are reconstructed into metric three-dimensional coordinates using calibrated stereo triangulation, providing frame-level trajectories and pose information. The dataset includes raw stereo video files, annotation files in CVAT XML format, processed three-dimensional trajectory data in CSV format, stereo calibration parameters, and scripts for reconstruction. Technical validation measures are provided to document dataset quality, including stereo calibration consistency assessed using stereo reprojection error of 0.50 pixels, annotation reliability analysis, and qualitative assessment of physically plausible motion patterns. By integrating synchronized stereo recordings, identity-consistent 2D annotations, and reconstructed 3D trajectories within a single resource, the dataset supports applications such as multi-object tracking, 3D pose estimation, trajectory modelling, and the analysis of multi-agent interaction, while detailed wing kinematic analysis is constrained by interpolation uncertainty and rolling-shutter effects.

Specifications Table

**Table:** 

Subject	Computer Sciences
Specific subject area	Stereo vision-based 3D reconstruction and multi-agent animal behaviour tracking.
Type of data	Table; Image; Video (MP4); Annotation files (XML); CSV files; Figures; Raw; Processed
Data collection	Data were collected using two SJCAM SJ4000 Air cameras (Sony IMX179 sensor) at 120 fps and 1280 × 720 resolution with a 0.69 m stereo baseline in an indoor flight arena. Cameras were hardware synchronized using an Arduino-based TTL trigger. Manual annotations were performed in CVAT using bounding boxes and keypoints. Stereo calibration was conducted in MATLAB using a checkerboard pattern, and 3D trajectories were reconstructed via triangulation.
Data source location	The data were collected at the Department of Computer Science, American International University-Bangladesh (AIUB), Kuratoli, Khilkhet, Dhaka 1229, Bangladesh (23.822028,90.427581).
Data accessibility	Repository name: Zenodo Data identification number: 10.5281/zenodo.19712127 Direct URL to data: https://doi.org/10.5281/zenodo.19712127 All data are publicly available via the Zenodo repository listed above.
Related research article	None

## 1 Value of the Data

• The dataset provides synchronized stereo videos with identity-consistent annotations and reconstructed three-dimensional trajectories, combining visual data and spatial coordinates in a single resource.
• It includes structured flight scenarios (solo, one-to-one, two-to-two, and three-to-three), providing examples of flight interactions under different group configurations.
• The dataset is primarily suited to multi-object tracking, body-trajectory analysis, and coarse 3D pose estimation as its main demonstrated use cases, while fine-scale wing kinematics and strong biological inference (e.g., collision-avoidance rules) are supported only as exploratory applications.
• The availability of paired two-dimensional annotations and three-dimensional reconstructions supports benchmarking and comparison of 2D-to-3D mapping approaches.
• The dataset includes raw videos, annotation files, calibration parameters, and processing scripts, allowing reproducibility and alternative processing workflows.
• The structured interaction scenarios may facilitate future analysis of behavioural descriptors such as path curvature, spatial separation, and group interaction dynamics during flight.

## 2 Background

Quantitative analysis of avian flight behaviour requires precise three-dimensional trajectory data from multiple interacting individuals [[1], [2], [3]]. Existing datasets include GPS-based flock tracking [1], controlled stereo recordings of individual flight behaviour [2,3], multi-view three-dimensional reconstruction frameworks [4,5], and detection-oriented datasets [6,7]. These datasets typically do not combine synchronized stereo geometry with identity-consistent annotations during multi-individual interactions.

Stereo vision systems enable three-dimensional reconstruction through calibrated camera triangulation, supporting spatial measurements [8,9]. High-speed video acquisition captures rapid motion, including wingbeats and short-duration interaction events [[10], [11], [12]]. Manual annotation with identity labels enables frame-level tracking of individuals during close-proximity interactions.

The dataset was generated using a calibrated stereo camera setup to record Budgerigar flights in a controlled indoor environment. Data collection includes solo flights and structured head-on interaction scenarios (one-to-one, two-to-two, and three-to-three configurations). Annotations were generated in both camera views using identity-consistent bounding boxes and keypoints, and corresponding three-dimensional coordinates were reconstructed using stereo triangulation. The dataset integrates recordings, annotations, calibration parameters, and reconstructed trajectories. This data article is released as a standalone dataset. However, the dataset is primarily intended for body-level trajectory reconstruction and multi-object tracking, while wing kinematics and strict cross-condition behavioural comparisons are limited by annotation interpolation and imaging constraints.

## 3 Data Description

Each recording contains approximately 320 frames, corresponding to approximately 2.5 ± 0.3 s per flight bout. All videos were manually annotated in both camera views using CVAT in interpolation mode. For each bird in every frame, an identity-consistent full-body bounding box and four single-point landmarks (head, tail, left wing, and right wing) were labelled. In multi-bird sequences, birds were consistently identified as B1–B6, with half of the birds representing one approaching group (e.g., B1–B2 in 2-to-2) and the remaining birds representing the opposing group. As summarized in Table 1, the dataset comprises 360 stereo video pairs across solo and multi-bird avoidance scenarios, with over 2.7 million annotated instances including bounding boxes and keypoints. This annotation scheme provides more detailed pose information than body-only labelling approaches commonly used in avian tracking datasets. Each of the 360 stereo recordings is therefore associated with corresponding files in the repository: two synchronized MP4 video files (Camera 1 and Camera 2), two CVAT XML annotation files (one per camera view), and one merged multi-bird 3D trajectory CSV file, yielding 720 MP4 files, 720 XML files, and 360 CSV files in total.

**Table 1:** Dataset composition: 360 stereo video pairs (100 solo + 260 avoidance trials), corresponding to 720 individual MP4 files across synchronized left/right camera streams, with manual annotations for multi-agent flight analysis.

Scenario	Total Stereo Video Pairs	Forward/ Return	Duration (s)	Birds/ video	XML Files	Total Boxes	Total Keypoints	Total Annotations
Solo Flight	100	50/50	2.5 ± 0.3	1	200	61,657	246,618	308,275
1-to-1 avoidance	150	75/75	2.5 ± 0.3	2	300	194,216	776,840	971,056
2-to-2 avoidance	80	40/40	2.5 ± 0.3	4	160	187,497	749,873	937,370
3-to-3 avoidance	30	15/15	2.5 ± 0.3	6	60	108,696	434,781	543,477
**Total**	**360**	**180/180**	**—**	**—**	**720**	**552,066**	**2208,112**	**2760,178**

Calibrated stereo triangulation implemented in MATLAB was used to reconstruct metric three-dimensional body centre and keypoint trajectories from the synchronized two-dimensional annotations, as summarized in Table 2. The resulting trajectories are provided as merged multi-bird CSV files for each video.

**Table 2:** Overview of processed trajectory CSV dataset. CSV files use wide format where each row represents one frame containing data for all birds in that frame (e.g., a 320-frame video with 4 birds contains 320 rows, where each row stores synchronized data for all four birds).

Scenario	Trajectory CSV Files	Total Data Rows	Features Dimensions	Total Data Points
Solo Flight	100	31,107	18	559,926
Avoidance (1-to-1)	150	49,997	35	1749,895
Avoidance (2-to-2)	80	24,321	69	1678,149
Avoidance (3-to-3)	30	9276	103	955,428
**TOTAL**	**360**	**114,701**	**—**	**4943,398**

The repository includes 360 stereo recordings, each consisting of two synchronized MP4 video files (1280 × 720 resolution, 120 frames per second, with an average duration of approximately 2.5 s), along with CVAT XML annotation files (separate files for Camera 1 and Camera 2), bird three-dimensional trajectory CSV files, stereo calibration parameters, and MATLAB processing scripts. All CSV files follow a consistent per-bird column schema, as detailed in Table 3. In merged multi-bird files, this schema is replicated for each bird using bird-specific prefixes (e.g., B1_Box_X, B2_Box_X).

**Table 3:** Columns in the processed 3D trajectory CSV files (Solo Flight: 18 columns; Avoidance scenarios: 35–103 columns). Note: Bounding box dimensions (Box_W, Box_H) are in pixels; all position coordinates are in millimetres.

Column	Description	Units
Frame	Frame index (0-based)	—
B1_Box_X, B1_Box_Y, B1_Box_Z	3D bounding box centre position	mm
B1_Box_W, B1_Box_H	Bounding box width and height	pixels
B1_Head_X, B1_Head_Y, B1_Head_Z	3D head coordinate	mm
B1_Tail_X, B1_Tail_Y, B1_Tail_Z	3D tail coordinate	mm
B1_LeftWing_X, B1_LeftWing_Y, B1_LeftWing_Z	3D left wing coordinate	mm
B1_RightWing_X, B1_RightWing_Y, B1_RightWing_Z	3D right wing coordinate	mm

**Fig. 1:**
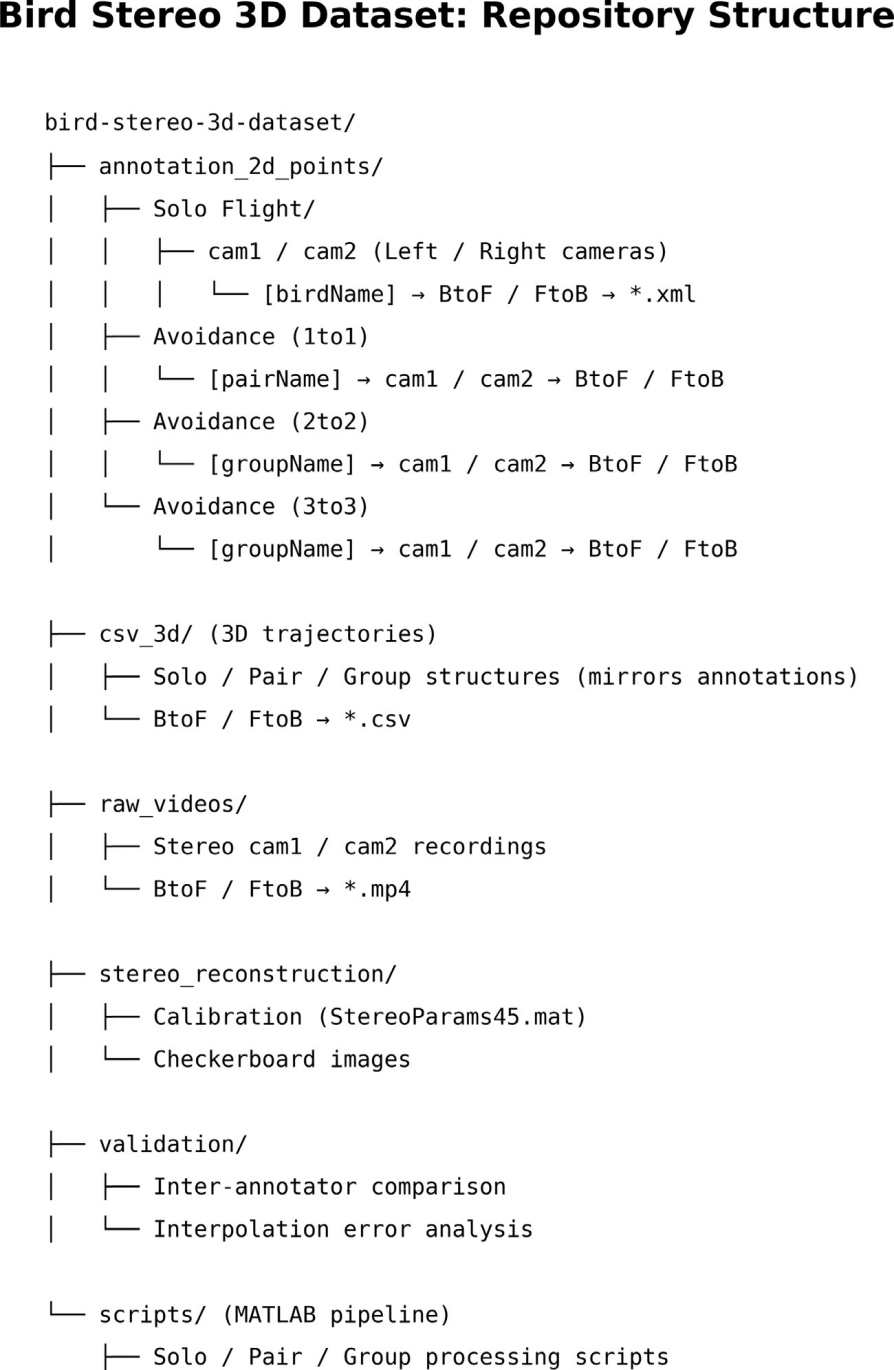
Overview of the dataset repository structure showing the organization of raw stereo videos, annotation files, calibration parameters, and processed three-dimensional trajectory data.

The dataset repository is organized into multiple structured directories (Fig. 1) to facilitate efficient access and reproducibility. Raw stereo video recordings are stored separately for each camera, along with corresponding annotation files in CVAT XML format. Processed data, including reconstructed three-dimensional trajectories in CSV format, are provided in merged multi-bird format. The repository also includes stereo calibration parameters and MATLAB scripts used for triangulation and data processing. This hierarchical organization ensures a clear separation between raw data, annotations, and processed outputs, enabling flexible use across computer vision, trajectory analysis, and behavioural research applications.

## 4 Experimental Design, Materials and Methods

### 4.1 Experimental setup

**Fig. 2:**
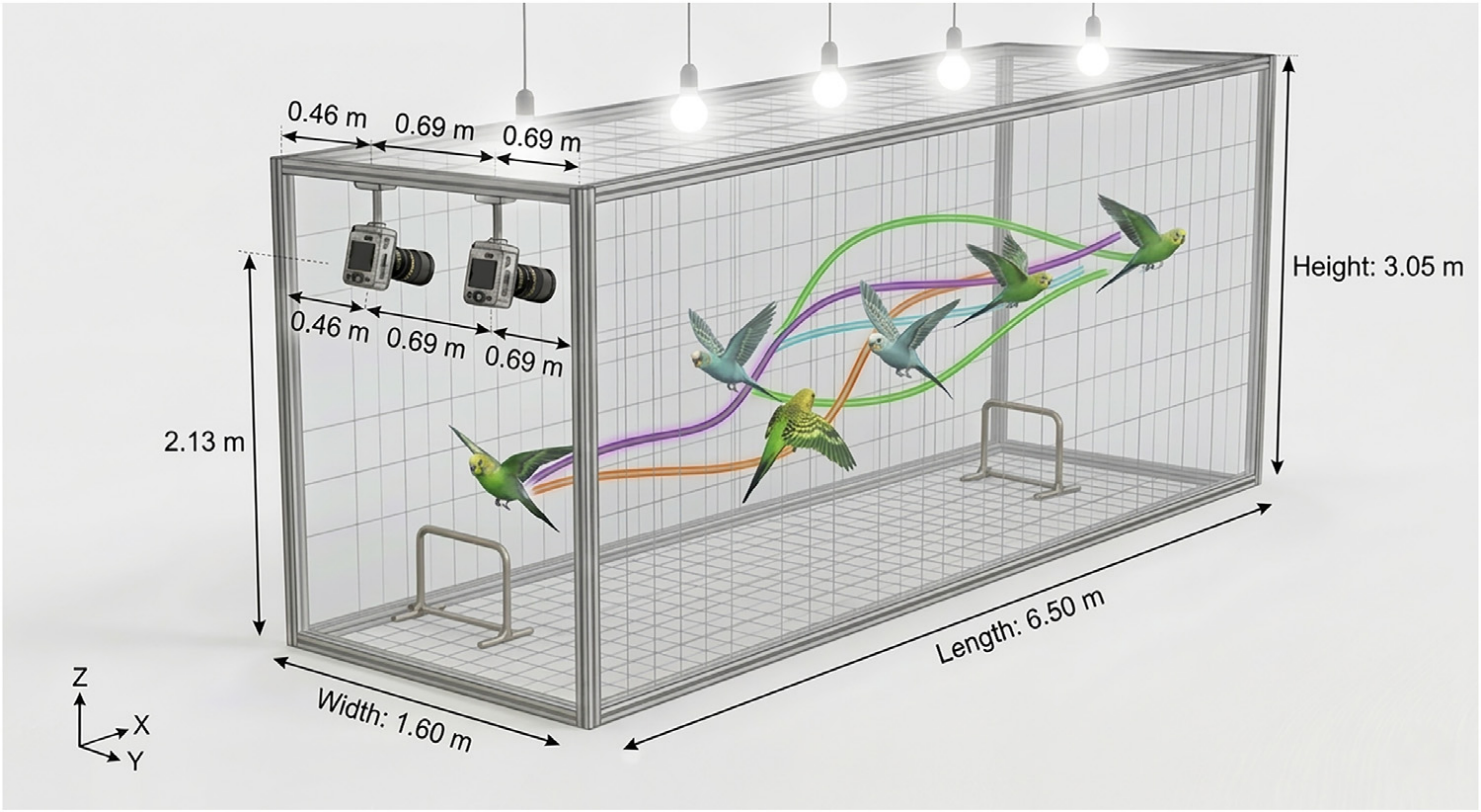
Experimental flight arena setup showing the 6.50 m arena length and stereo camera configuration with a 0.69 m baseline. This figure is a schematic illustration and not a scale-accurate representation of the experimental setup.

Experiments were conducted in a controlled indoor flight arena measuring 6.50 m × 1.60 m × 3.05 m (Fig. 2). Interior surfaces were covered with matte black cloth to minimize reflections and enhance contrast.

Illumination was provided by five 300 W warm white filament bulbs (100 Hz AC supply) to minimize flicker artifacts during 1/2000 s shutter speed capture. No measurable flicker was observed. Two identical SJCAM SJ4000 Air cameras (Sony IMX179 sensor) were mounted at 2.13 m height with 0.69 m horizontal baseline, slightly converged to maximize stereo overlap in the central flight corridor (5 m × 1.2 m × 2.5 m). Videos were recorded at 1280 × 720 resolution at 120 fps with 1/2000 s shutter speed. Hardware synchronization was implemented using an Arduino-based TTL trigger that simultaneously initiated video acquisition on both cameras through a common external trigger pulse. Because the cameras operated at a fixed frame rate after simultaneous triggering, corresponding frames from both video streams remained temporally aligned throughout each recording sequence.

Elevated landing platforms (bird cages on tables at 0.9 m height) at opposite ends encouraged voluntary flights along the arena’s longitudinal axis.

The study subjects comprised ten adult Budgerigars (Melopsittacus undulatus), a small parrot species native to Australia (Table 4). The cohort included eight males (Earth, Jupiter, Mars, Mercury, Neptune, Pluto, Saturn, and Uranus) and two females (Eris and Venus). All birds were approximately one year old, flight-trained, and housed in a communal aviary when not participating in experiments. They were maintained in good health with ad libitum access to food and water.

**Table 4:** Subject demographics: ten adult Budgerigars (eight males, two females) with approximate body mass and wingspan measurements.

Bird Name	Sex	Age (years)	Mass (g)	Wingspan (cm)	Identifying Feature
Earth	Male	1	∼29	∼29	White head
Jupiter	Male	1	∼28	∼29	Black wing tip
Mars	Male	1	∼32	∼28	Ash tail
Mercury	Male	1	∼33	∼32	Yellow head
Neptune	Male	1	∼35	∼28	Green belly, black tail
Pluto	Male	1	∼28	∼27	Blue tail
Saturn	Male	1	∼33	∼31	Bright green body
Uranus	Male	1	∼32	∼32	Light green body
Eris	Female	1	∼27	∼27	Cyan body, black tail
Venus	Female	1	∼28	∼28	Yellow body

### 4.2 Camera Calibration, Synchronization, and 3D Reconstruction

Stereo calibration was performed in MATLAB R2024a [13] using the Stereo Camera Calibrator application. We used a planar 9 × 7 checkerboard pattern (square size: 60 mm) for calibration. Each calibration image contained 48 detectable inner corner points (8 × 6 grid).

**Fig. 3:**
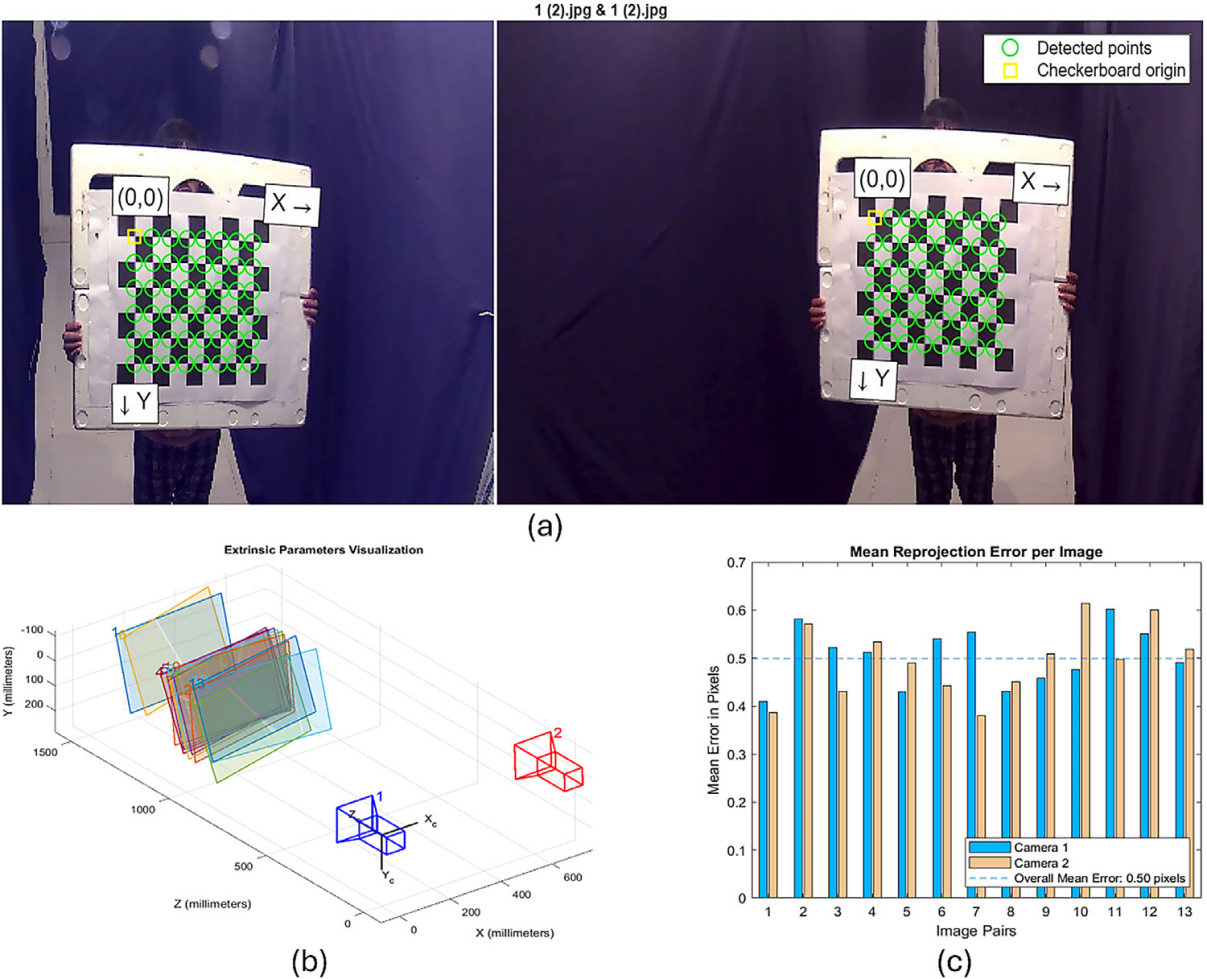
Stereo camera calibration using checkerboard pattern across 13 viewpoints spanning the flight volume.

A total of 13 stereo image pairs were captured at varying distances (0.8–4.5 m) and orientations throughout the usable flight volume (Fig. 3**a**). Checkerboard positions were distributed to cover the full arena depth range and lateral extents (Fig. 3**b**): 3 positions at 0.8–1.5 m depth near the cameras, 5 positions at 2.0–3.5 m in the mid-corridor, and 5 positions at 3.5–5.0 m in the far corridor, with lateral offsets covering ±0.6 m from corridor centre. This resulted in 624 valid corner detections per camera (13 × 48), corresponding to 1248 stereo corner correspondences.

The mean stereo reprojection error was 0.50 pixels (root mean square) (Fig. 3**c**), showing subpixel calibration accuracy.

All intrinsic and extrinsic calibration parameters are provided in the .mat file within the data repository to ensure full reproducibility of the 3D reconstruction.

Temporal synchronization between cameras was achieved using a common external hardware trigger (Arduino-based TTL pulse), ensuring frame-level alignment across the stereo pair. Synchronization accuracy was verified using simultaneous high-speed LED flashes visible in both camera views, after which the calibrated parameters were used to undistort and rectify all video frames prior to triangulation. For each annotated frame, 2D keypoints from both camera views were triangulated into metric 3D coordinates using MATLAB’s triangulate function with the calibrated stereo parameters. The body centre was defined as the 3D midpoint of the reconstructed bounding box, providing a stable longitudinal body axis reference independent of wing flapping dynamics.

To ensure consistency across trajectories and facilitate comparative analysis, all reconstructed 3D coordinates were transformed from the original stereo camera coordinate system into a tunnel-aligned world coordinate system using a rigid-body transformation. First, a fixed origin offset of [−28,931.846, −18,530.889, 45,538.043] mm was subtracted from all reconstructed points to shift the coordinate origin from the camera optical centre toward the physical flight corridor. Subsequently, a rotation matrix derived from three reference points at known physical locations within the arena (the two landing platforms and the corridor centre) was applied to align the coordinate axes with the tunnel geometry. The transformed coordinate system follows a right-handed convention in which the X-axis represents the longitudinal flight direction (forward along the tunnel), the Y-axis corresponds to the lateral direction (left-right), and the Z-axis denotes the vertical direction (positive upward). Because the transformation consists only of translation and rotation (without scaling or shear), the original metric structure, inter-point distances, and trajectory geometry are preserved. The numerical values of the translation offset and rotation matrix used for this transformation are provided in the calibration parameter file (StereoParams45.mat) and the accompanying MATLAB processing scripts in the data repository to ensure full reproducibility of the world-coordinate alignment.

### 4.3 Data collection and flight trials

Flight trials were designed to represent four levels of social complexity: (i) solo flight, (ii) one-to-one (1-to-1) head on avoidance, (iii) two-to-two (2-to-2) avoidance, and (iv) three-to-three (3-to-3) avoidance. For each condition, flights were recorded in two balanced directions: forward flights (approximately perpendicular to the primary camera axis) and return flights (opposite direction), ensuring comparable representation of approach and recession trajectories across experimental conditions (Table 1).

In solo trials, a single bird was released from one elevated landing platform and voluntarily flew to the opposite platform. Flights were encouraged using mild food reward and conditioned hand signals, without physical coercion.

In avoidance trials (1-to-1, 2-to-2, and 3-to-3), birds were released from opposite platforms such that they naturally approached each other within the central flight corridor. Take-off occurred voluntarily after recording onset, with birds typically initiating flight within 200 ms on average. No physical barriers or external obstacles were introduced; therefore, all avoidance manoeuvres resulted solely from visual interaction with oncoming conspecifics.

**Fig. 4:**
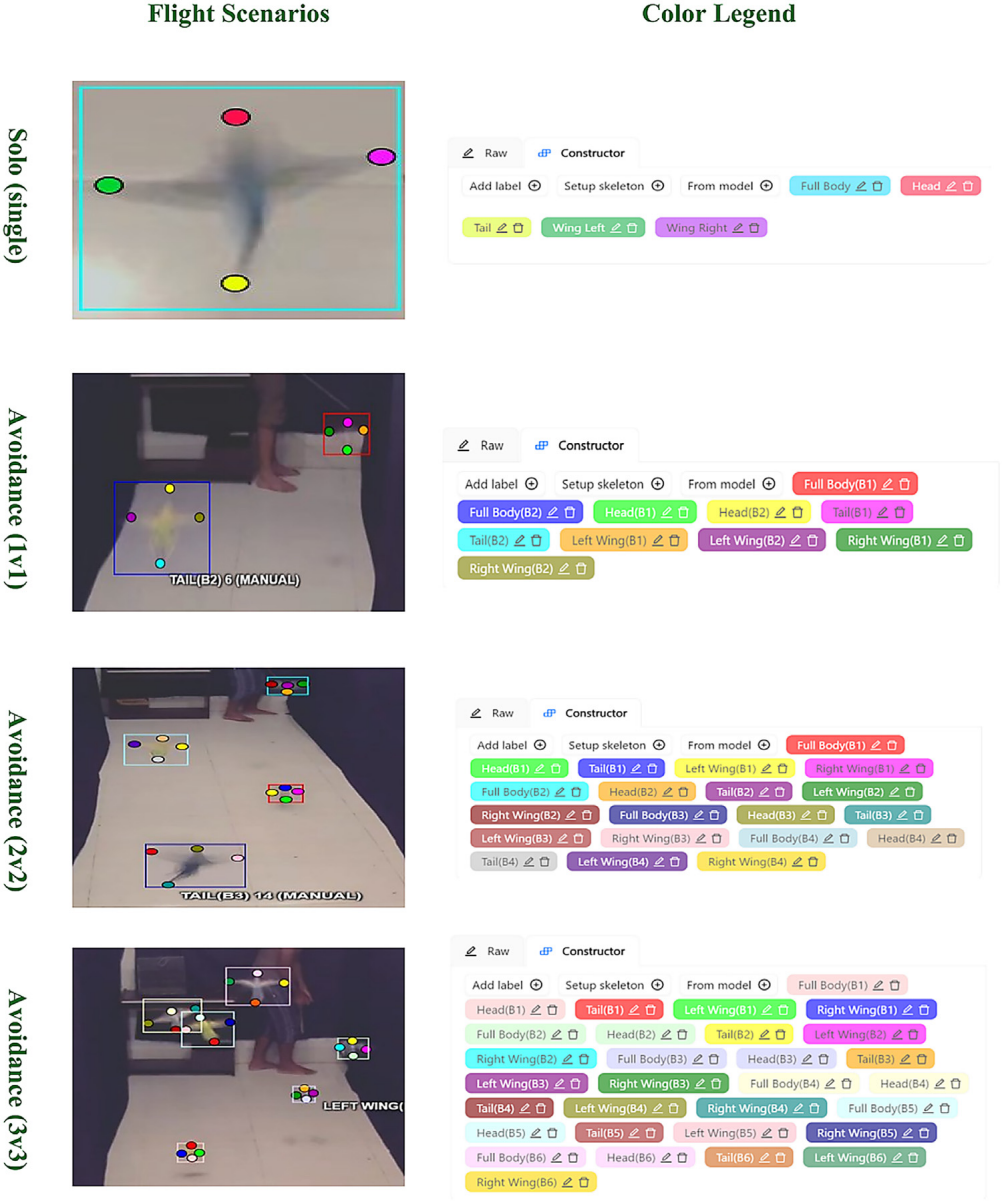
Representative annotated video frames showing (a) solo flight, (b) 1-to-1 head-on encounter, (c) 2-to-2 group avoidance, and (d) 3-to-3 multi-bird interaction with identity-consistent bounding boxes and body keypoints.

Only trials exhibiting a complete behavioural sequence — clear take-off, mid-flight interaction within the central corridor, and successful landing — were retained for the dataset (Fig. 4). The final dataset comprised 100 solo flights, 150 one-to-one trials, 80 two-to-two trials, and 30 three-to-three trials, resulting in a total of 360 stereo video pairs.

Stereo annotations from Camera 1 and Camera 2 were frame synchronized between the first and last annotated frames. In rare instances where one camera recorded a few additional frames due to minor trigger variation, excess frames at the beginning or end of the longer sequence were removed. This ensured that both camera views covered identical temporal intervals and corresponding bird pose across perspectives.

A total of 986 stereo flight trial attempts were recorded across all experimental conditions, of which 360 stereo video pairs were retained in the final dataset. Trials were excluded when they did not satisfy the predefined completeness criteria consisting of: (i) voluntary take-off, (ii) observable mid-flight interaction within the central stereo overlap corridor, and (iii) successful landing within the recording interval. Excluded trials primarily resulted from one or more of the following conditions: birds failing to initiate flight after recording onset, incomplete entry into the stereo overlap region, partial or complete exit from the camera field of view, excessive inter-bird occlusion preventing reliable annotation, unsuccessful synchronization of simultaneous take-off in multi-bird conditions, or incomplete landing behaviour before recording termination. Most exclusions occurred in the 3-to-3 condition, where coordinating six birds to voluntarily initiate synchronized opposing flights proved substantially more difficult than in lower-density conditions. In many attempted trials, one or more birds delayed take-off, deviated from the central corridor, or landed prematurely, preventing consistent mid-flight interaction between the two opposing groups. Consequently, the final dataset preferentially consists of trials exhibiting complete and visually trackable interaction sequences, prioritizing annotation quality and stereo reconstruction consistency over balanced sampling across conditions.

### 4.4 Video annotation and 3D trajectory generation

**Fig. 5:**
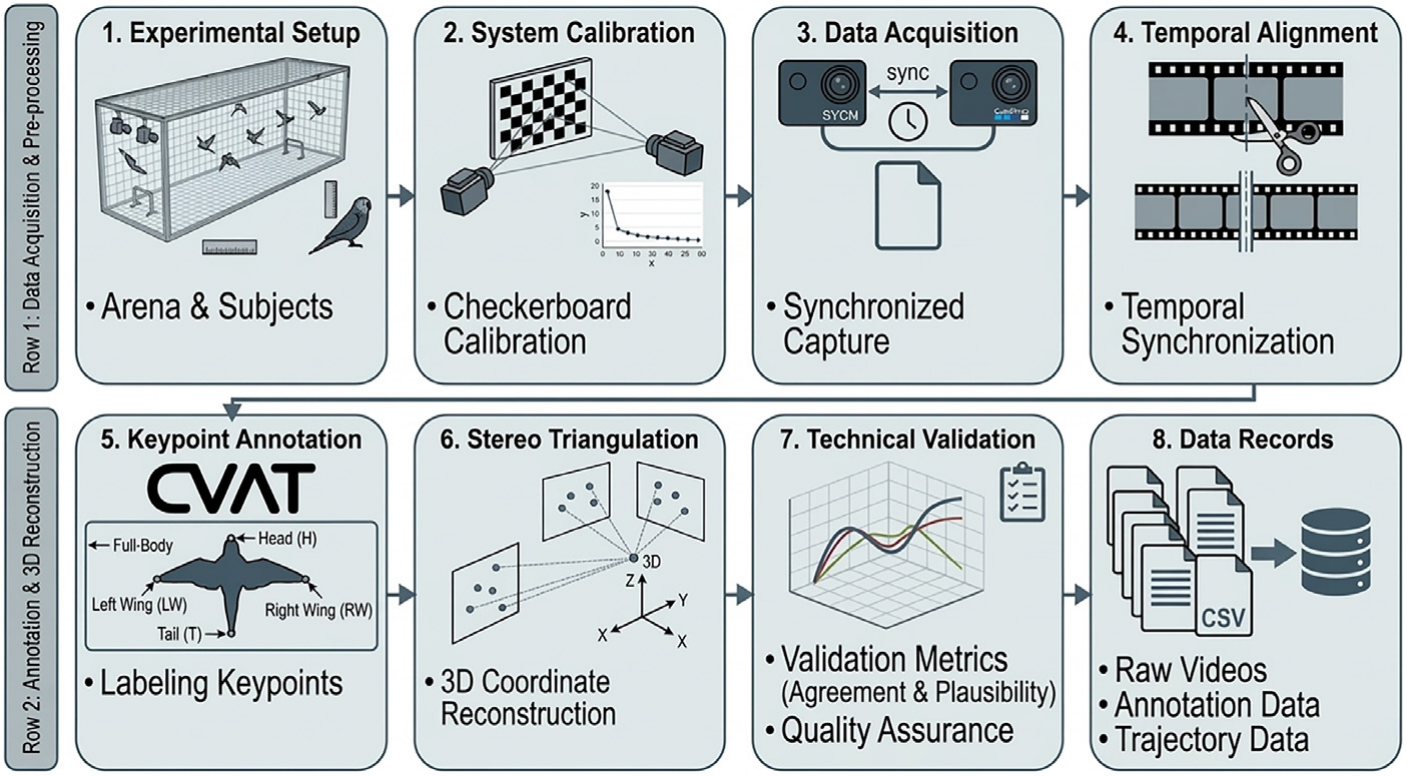
Annotation workflow and 3D reconstruction pipeline. This figure is a schematic illustration.

Synchronized stereo video pairs were imported into CVAT (version 2.10.1) [14] and configured as interpolation tasks. For each bird, five annotation types were defined: (i) full-body axis-aligned bounding box, and four single-point keypoints corresponding to (ii) head, (iii) tail, (iv) left wing, and (v) right wing (Fig. 5).

In multi-bird sequences, individuals were assigned persistent identity labels (B1–B6). Birds released from one platform were indexed consecutively (e.g., B1–Bn), while birds released from the opposite platform were assigned subsequent indices. Identity consistency was maintained throughout each video sequence.

In multi-bird sequences, individuals were assigned persistent identity labels (B1–B6). Birds released from one platform were indexed consecutively (e.g., B1–Bn), while birds released from the opposite platform were assigned subsequent indices. Identity consistency was maintained throughout each video sequence by visually tracking each bird's distinctive plumage features (e.g., head colour, wing-tip markings, and tail colour; see Table 4) frame by frame during annotation. In the rare cases of close crossings or partial occlusion where automatic continuity was ambiguous, the annotator paused playback, compared the bird's identifying features across consecutive frames in both camera views, and manually corrected any identity switches before continuing. Because all ten birds had clearly distinguishable visual markers, identity assignment could be verified directly from the video content without relying on additional tracking algorithms.

A trained annotator manually inserted keyframes at fixed intervals of every fourth frame throughout all video sequences. Specifically, one frame was manually annotated followed by three interpolated frames generated using CVAT’s linear interpolation function (i.e., Manual → Interpolated → Interpolated → Interpolated → Manual). This protocol was applied consistently across the entire dataset for both camera views. Consequently, manually annotated frames can be identified directly from the frame index in the CVAT XML files, as every fourth frame (starting from the first frame of each sequence) corresponds to a manual annotation, while the three intermediate frames between consecutive manual annotations correspond to linearly interpolated values. This allows users to filter, weight, or exclude interpolated frames according to the requirements of their downstream analysis. Between keyframes, bounding boxes and keypoints were linearly interpolated using CVAT’s built-in interpolation function. Identical keyframe indices were applied to both camera views to preserve temporal alignment across the stereo pair. Interpolation accuracy varied across anatomical landmarks and was lower for wing keypoints than for head or body-centre locations. To assess interpolation consistency, a dataset-wide leave-one-out evaluation was performed using the existing annotation structure, in which manually annotated keyframes were temporarily withheld and reconstructed using neighbouring manual frames. This evaluation measures internal reconstruction consistency using existing manual annotations rather than requiring additional external ground-truth data. As summarized in Table 5, wing keypoints exhibited comparatively larger interpolation deviations, likely due to rapid wingbeat motion and partial occlusion. Interpolation deviations were lower for head and body-centre locations than for wing keypoints. Therefore, the dataset may be more suitable for trajectory-level analysis than for fine-scale wing kinematic analysis.

**Table 5:** Interpolation consistency analysis using dataset-wide leave-one-out evaluation across key anatomical landmarks.

Keypoint	n	Mean ± SD (px)
Head	67,898	3.68 ± 8.51
Tail	69,485	5.24 ± 8.28
Left Wing	72,891	11.51 ± 12.54
Right Wing	72,884	11.51 ± 12.56
Body bounding-box centre	67,942	4.35 ± 8.73

Note: Sample sizes (n) differ across keypoints due to frame-level visibility and reconstruction validity constraints in the leave-one-out evaluation; only manually annotated frames with successful stereo visibility were included, and minor differences arise from numerical filtering during reconstruction.

When a bird was fully occluded (>90% bounding box overlap), no annotation was placed. For partial occlusion (e.g., wing behind body or bird behind conspecific), visible keypoints were annotated while occluded keypoints were marked as outside image bounds. Because wing regions were more frequently affected by rapid motion and partial occlusion during mid-wingbeat phases, interpolation deviations for wing keypoints were generally higher than for head or body-centre locations. Three-dimensional triangulation used only mutually visible keypoints; frames with fewer than two camera views available for a keypoint produced null coordinates in the CSV files (marked as NaN).

3D reconstruction was performed as described in the previous section. The resulting 3D trajectories were exported as merged multi-bird CSV files, with one CSV file provided per stereo recording in wide format containing frame-synchronized trajectory data for all individuals. Accordingly, the dataset includes 360 merged trajectory CSV files in total. Solo flight CSV files contain 18 columns (1 bird), 1-to-1 avoidance files contain 35 columns (2 birds), 2-to-2 files contain 69 columns (4 birds), and 3-to-3 files contain 103 columns (6 birds).

In addition to interpolation-related uncertainty, the rolling-shutter architecture of the SJCAM SJ4000 Air cameras (approximately 18 ms full-frame readout) may introduce motion distortion during rapid wing movement. At representative flight speeds, fast-moving wingtip structures may experience measurable geometric distortion, potentially affecting high-precision biomechanical measurements. Combined with fixed keyframe spacing and linear interpolation, this limitation primarily affects fine-scale wing kinematic analyses more than body-centre or trajectory-level applications. This fixed interpolation strategy provides practical scalability across large datasets but may reduce precision for rapidly moving anatomical landmarks, particularly wing keypoints, where linear interpolation may not fully capture high-frequency motion dynamics. Dataset-wide leave-one-out analysis confirmed lower interpolation error for head and body-centre coordinates than for wing landmarks, reinforcing that the dataset is more robust for trajectory reconstruction, tracking, and coarse pose estimation than for detailed wingbeat biomechanics.

### 4.5 Stereo calibration accuracy

Stereo calibration was assessed using the MATLAB Stereo Camera Calibrator toolbox. Across 13 stereo checkerboard image pairs (48 inner corners per image), the mean reprojection error was 0.50 pixels, indicating stable subpixel stereo calibration performance within the usable flight corridor. Based on the calibrated stereo geometry and image scale at a median working distance of approximately 3 m, the effective spatial sensitivity was estimated to be on the order of 0.4–0.5 mm/pixel under ideal triangulation conditions. The corresponding 3D localization uncertainty in the central corridor is expected to be on the order of a few millimetres in the lateral directions, with somewhat higher uncertainty along the depth axis that scales with object distance from the cameras. This value should be interpreted as a theoretical order-of-magnitude estimate derived from stereo geometry rather than a direct empirical measurement, because no independent ground-truth 3D reference object or motion-capture system was available for absolute validation across the flight volume.

Actual reconstruction error may therefore vary with depth, viewing angle, rolling-shutter distortion, annotation uncertainty, partial occlusion, and residual temporal synchronization uncertainty between stereo views, particularly near corridor boundaries or during rapid motion. Calibration robustness was verified by excluding points with reprojection error > 1 pixel or depth outside arena boundaries (< 1% of points excluded). Axis-wise reconstruction error (X, Y, Z) was not empirically quantified because no external ground-truth measurement system was available during acquisition. Users should therefore interpret reconstructed coordinates with caution, particularly near corridor boundaries or under rapid motion and occlusion conditions.

### 4.6 Annotation consistency

Inter-annotator reliability was evaluated by independent re-annotation of 20 representative video clips spanning all experimental conditions (Solo: 5, 1-to-1: 5, 2-to-2: 5, 3-to-3: 5) by a second trained annotator who had no access to the original annotations during the re-annotation. For each clip, 1–2 representative frames were re-annotated, yielding 66 paired bird-frame instances in total. For every paired sample, the Euclidean pixel distance between the two annotators' head, tail, left wing, and right wing keypoints was computed, while bounding-box agreement was quantified using both centre-point displacement and intersection-over-union (IoU). The resulting inter-annotator reliability metrics are summarized in Table 6.

**Table 6:** Inter-annotator reliability metrics for 2D keypoint annotations across 66 paired bird–frame instances from 20 video clips.

Metric	n	Mean ± SD	Median	IQR	PCK@5	PCK@10
Head	66	5.21 ± 2.78 px	5.15 px	3.43 px	45.5%	95.5%
Tail	66	6.43 ± 3.55 px	5.50 px	5.66 px	47.0%	81.8%
Left Wing	66	12.94 ± 6.83 px	11.46 px	9.34 px	10.6%	39.4%
Right Wing	66	12.30 ± 6.14 px	11.86 px	8.32 px	9.1%	40.9%
Bounding-box centre MAE	66	1.97 ± 1.02 px	2.01 px	1.24 px	—	—
Bounding-box IoU	66	0.888 ± 0.056	0.891	0.078	—	—

Head and tail keypoints showed lower mean errors (5.21 ± 2.78 px and 6.43 ± 3.55 px, respectively) than wing keypoints (12.94 ± 6.83 px and 12.30 ± 6.14 px), in line with the higher motion dynamics and occlusion sensitivity of wing landmarks during flight. Median errors were generally lower than the corresponding means, suggesting right-skewed distributions in which a portion of frames containing rapid wing motion or partial occlusion contributed to the heavier tails. The percentage of correct keypoints (PCK) followed the same trend: head and tail annotations achieved PCK@10 values of 95.5% and 81.8%, indicating that the majority of these landmarks were localized within 10 pixels across annotators, whereas wing landmarks reached only 39.4% (left) and 40.9% (right) at the same threshold, reflecting their greater positional uncertainty. At the stricter PCK@5 threshold, head and tail keypoints retained moderate agreement (45.5% and 47.0%), while wing keypoints dropped to about 10%, further highlighting the limited fine-scale precision for wings. Bounding-box annotations showed reasonable agreement, with a mean centre displacement of 1.97 ± 1.02 px (median 2.01 px, IQR 1.24 px) and a mean IoU of 0.888 ± 0.056 (median 0.891, IQR 0.078). These results indicate that the dataset is suitable for trajectory-level analysis, multi-object tracking, and coarse pose estimation, while fine-scale wing kinematic analyses should account for the comparatively higher uncertainty in wing keypoint annotations. The reliability assessment was conducted on Camera 1 views as a representative sample; both camera views employed an identical annotation protocol, software, and annotators.

### 4.7 Temporal synchronization

Frame-level synchronization between the stereo cameras was achieved using a shared Arduino-based TTL trigger that simultaneously initiated acquisition on both cameras. Following triggering, both cameras operated at a fixed acquisition rate of 120 fps, resulting in synchronized frame progression throughout each recording.

Synchronization consistency was verified using repeated high-speed LED flash tests visible simultaneously in both camera views prior to experimental recording. A total of 50 synchronization verification recordings were inspected. For each test, the frame index corresponding to LED onset was compared between Camera 1 and Camera 2. In all inspected LED tests, the LED onset appeared either in the same frame or within a maximum discrepancy of one frame (8.3 ms at 120 fps), with most tests showing exact frame correspondence.

Additional qualitative verification was performed by visually comparing rapid wingbeat phases across stereo views during flight sequences, which showed temporally consistent motion patterns without observable systematic drift over recording duration.

A residual synchronization uncertainty of up to one frame may introduce additional triangulation uncertainty during rapid wing motion or high-speed lateral displacement. Assuming a representative flight speed of approximately 2 m/s, a worst-case temporal offset of 8.3 ms corresponds to an apparent positional displacement of approximately 16–17 mm. However, because most synchronized frames exhibited identical LED timing and the synchronization error was not systematic across recordings, the practical impact on reconstructed body-centre trajectories is expected to be substantially smaller for most frames. Users performing high-precision wing kinematic analysis should consider this residual temporal uncertainty when interpreting rapidly moving wingtip coordinates.

### 4.8 Biological plausibility of 3D trajectories

**Fig. 6:**
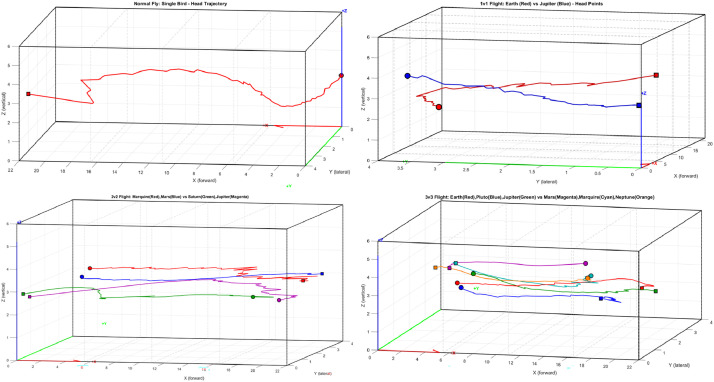
Representative 3D trajectory reconstructions across solo and multi-bird avoidance conditions. Start (circle) and end (square) markers indicate flight direction from perch to perch.

Reconstructed trajectories were qualitatively consistent with expected Budgerigar flight behaviour, exhibiting visually consistent flight trajectories (Fig. 6). Solo flights were characterized by approximately linear trajectories with modest vertical displacement during take-off and landing phases.

In one-to-one trials, birds exhibited observable lateral or vertical trajectory deviations during head-on encounters, with minimum inter-individual distances typically ranging from 0.4 to 0.8 m. In multi-bird (2-to-2 and 3-to-3) trials, trajectories showed increased path curvature and spatial separation tendencies between opposing groups.

Across all trials, cruising flight speeds (excluding take-off and landing phases) ranged from approximately 1.6 to 2.0 m/s, with a mean speed of 1.8 ± 0.2 m/s (n = 360). Speed distribution was approximately normal (skewness = 0.15) and consistent across conditions: Solo (1.8 ± 0.2 m/s), 1-to-1 (1.8 ± 0.2 m/s), 2-to-2 (1.7 ± 0.2 m/s), and 3-to-3 (1.7 ± 0.2 m/s). These speeds are consistent with previously reported values for Budgerigars in confined flight environments and reflect the mid-flight cruising phase within the 3–4 m central corridor.

Minimum inter-bird distances during head-on encounters ranged from 0.4 to 0.8 m, with a mean of 0.6 ± 0.1 m (n = 260 avoidance trials). Distance distribution was slightly right-skewed (skewness = 0.45), with 85% of encounters achieving minimum separation greater than 0.5 m. The distribution varied by condition: 1-to-1 (mean 0.6 ± 0.1 m), 2-to-2 (mean 0.6 ± 0.1 m), and 3-to-3 (mean 0.5 ± 0.1 m), reflecting increased spatial constraints in larger groups. The presented analyses are intended primarily to verify reconstruction consistency and trajectory plausibility rather than to establish definitive behavioural interaction models.

### 4.9 Usage notes

Users should note that bounding box width and height columns (Box_W, Box_H) are stored in pixels, while all position data are stored in millimetres. For consistent metric analysis, multiply Box_W and Box_H by the approximate pixel scale (about 1.5 mm/pixel at mid-corridor distance), or use only Box_X, Box_Y, and Box_Z for spatial measurements.

For trajectory analysis, users are advised to use the head coordinates (Head_X, Head_Y, Head_Z) as a stable proxy for body position. The head point provides a reliable trajectory reference because it is less affected by body oscillations and wing motion than other keypoints. Processed CSV files can be directly imported into Python, MATLAB, or similar numerical computing environments for trajectory analysis, with native plotting tools available in both platforms.

Given the condition imbalance and higher interpolation uncertainty for wing keypoints, we recommend using the dataset primarily for trajectory-level and tracking applications and treating fine-scale wingbeat or cross-condition behavioural analyses as exploratory.

### 4.10 Reuse potential

This dataset can be used for behavioural ecology, computer vision, and bio-inspired robotics. The dataset is suited to body-trajectory analysis, multi-object tracking, and coarse 3D pose estimation. Applications requiring fine-scale wingbeat kinematics or balanced statistical comparisons across all group configurations (particularly involving the 3-to-3 condition) should be interpreted as exploratory. In behavioural ecology, the identity-consistent 3D trajectories may support future quantitative analysis of close-proximity flight interaction behaviour, group coordination dynamics, and interaction forces in flying birds. Researchers may use the dataset to explore motion descriptors such as minimum-distance distributions and path curvature under different interaction conditions.

In the domain of multi-agent tracking and 3D pose estimation, the stereo-synchronized annotated video pairs together with reconstructed 3D coordinates provide a benchmark for developing and validating multi-object tracking algorithms, 3D pose estimation methods, and depth-from-stereo techniques for fast-moving birds.

For trajectory prediction and modelling, the structured encounter scenarios (1-to-1, 2-to-2, 3-to-3) may be useful for studying interaction-aware trajectory modelling approaches. In addition, the dataset may inform bio-inspired navigation research, where observed avoidance manoeuvres can provide reference trajectories that may be relevant for future bio-inspired navigation or multi-agent motion studies.

The dataset is released under the CC-BY-4.0 license, permitting unrestricted academic and commercial reuse with proper attribution. All code for 3D reconstruction and trajectory processing is provided to facilitate reproducibility and extension of the work.

## Limitations

The dataset is imbalanced across conditions, with substantially fewer 3-to-3 trials than 1-to-1 trials because synchronized multi-bird take-off and stable mid-flight interaction were difficult to achieve consistently. Consequently, the dataset is more appropriate for exploratory behavioural analysis and trajectory modelling than for balanced statistical comparison across all conditions. Rolling-shutter effects and interpolation between manually annotated keyframes may introduce additional uncertainty during rapid wing motion, particularly for wingtip coordinates. Preliminary evaluation indicated lower interpolation consistency for wing keypoints compared with head or body-centre locations.

Absolute 3D reconstruction accuracy was not independently validated using an external motion-capture or surveyed ground-truth system. Therefore, the reported localization uncertainty should be interpreted as an approximate theoretical estimate derived from stereo geometry and image scale rather than a direct empirical measurement. Reconstruction uncertainty may increase near the stereo overlap boundaries, during rapid motion, or under partial occlusion. Although hardware triggering was used for synchronization, a residual temporal mismatch of up to one frame may still occur, which could introduce additional triangulation error for fast-moving body parts. Overall, the dataset is suited for trajectory-level analysis, multi-object tracking, and coarse 3D pose estimation.

## Ethics Statement

The Institutional Review Board (IRB) of American International University-Bangladesh (AIUB) reviewed and approved all animal experimental procedures (Protocol No: AIUB-IRB-FST-2026–02–01). The study was conducted in accordance with the ARRIVE guidelines and internationally accepted standards for the care and use of animals in research, including relevant institutional and national ethical regulations.

The data were obtained through non-invasive behavioural observation of adult Budgerigars (Melopsittacus undulatus). Birds were allowed to fly voluntarily within an indoor flight tunnel between two perches during brief recording sessions (2–3 h per day). At no point were the birds subjected to physical restraint, surgical intervention, chemical treatment, or aversive behavioural conditioning.

The sex of the animals was not explicitly controlled; however, no sex-specific experimental manipulation was performed, and no sex-related effects were analysed in this study.

The study did not involve human participants, human biological materials, social media data, dangerous chemicals, infectious agents, or genetically modified organisms. All data collection, processing, storage, and public release were conducted in accordance with ethical standards of animal welfare, data integrity, and responsible research practice.

## CRediT Author Statement

**S.M. Tawhid:** Conceptualization, Data curation, Formal analysis, Investigation, Methodology, Software, Validation, Visualization, Resources, Writing– original draft. **Abdul Kader Mohim:** Conceptualization, Data curation, Formal analysis, Investigation, Methodology, Software, Validation, Writing– review & editing. **Sk. Shahed Ali:** Conceptualization, Data curation, Formal analysis, Investigation, Methodology, Software, Validation, Writing– review & editing. **Kazi Tanzizul Haque Tanzil:** Conceptualization, Data curation, Investigation, Methodology, Validation, Writing– review & editing. **Dip Nandi:** Conceptualization, Investigation, Methodology, Validation, Writing– review & editing. **Abhijit Bhowmik:** Conceptualization, Data curation, Investigation, Methodology, Project administration, Resources, Supervision, Validation, Writing– review & editing. **Dr. Debajyoti Karmaker:** Conceptualization, Methodology, Project administration, Resources, Supervision, Writing– review & editing.

## Declaration of Generative AI and AI-Assisted Technologies in the Manuscript Preparation Process

During the preparation of this work, the authors used Google Gemini to assist in the preparation of schematic diagrams (Fig. 2, Fig. 5) and for minor language polishing. The use of this tool was limited to visualization support and language refinement. After using this tool/service, the authors carefully reviewed, verified, and edited all generated content as needed, and take full responsibility for the content of the published article. All scientific content, including the methodology, analysis, and results, was developed independently by the authors. The figures presented in this manuscript are based entirely on the authors’ original concepts, experimental design, and data. No AI-generated scientific content was used in the development of the study.

## Data Availability

ZenodoA Stereo Camera Dataset of Annotated Budgerigar Flight Trajectories for Studying Avoidance Behaviors in Multi-agent Scenarios (Original data).
